# Coexistence of two sympatric cryptic bat species in French Guiana: insights from genetic, acoustic and ecological data

**DOI:** 10.1186/s12862-018-1289-8

**Published:** 2018-11-20

**Authors:** Ondine Filippi-Codaccioni, Marie-Pauline Beugin, Damien M. de Vienne, Elodie Portanier, David Fouchet, Cecile Kaerle, Lina Muselet, Guillaume Queney, Eric J. Petit, Corinne Regis, Jean-Baptiste Pons, Dominique Pontier

**Affiliations:** 1 University Lyon, Université Lyon 1, CNRS, Laboratoire de Biométrie et Biologie Evolutive UMR5558, F-69622 Villeurbanne, France; 2 Université de Lyon, LabEx Ecofect, Nadine Cizaire, 92 rue Pasteur, CS 30122 69361 Lyon Cedex 07, France; 3 SEISE 26 bis Barrouil, 33720 Illats, France; 4 ANTAGENE, Animal Genomics Laboratory, 6 allée du Levant, 69890 La Tour de Salvagny (Lyon), France; 5 Université de Lyon, VetAgro Sup - Campus Vétérinaire de Lyon, 1 Avenue Bourgelat, BP 83, F-69280 Marcy l’Etoile, France; 6 Office National de la Chasse et de la Faune Sauvage - Unité Faune de Montagne, 147 Route de Lodève, Les Portes du Soleil, F-34990 Juvignac, France; 7 UMR ESE, Ecology and Ecosystem Health, INRA, Agrocampus Ouest, 65 rue de Saint-Brieuc, 35042 Rennes Cedex, France

**Keywords:** *Pteronotus* cf. *parnellii*, Microsatellite, Mitochondrial DNA, Phylogeny, Echolocation, Population genetics, Asymmetric introgression, Neotropics

## Abstract

**Background:**

The distinction between lineages of neotropical bats from the *Pteronotus parnellii* species complex has been previously made according to mitochondrial DNA, and especially morphology and acoustics, in order to separate them into two species. In these studies, either sample sizes were too low when genetic and acoustic or morphological data were gathered on the same individuals, or genetic and other data were collected on different individuals. In this study, we intensively sampled bats in 4 caves and combined all approaches in order to analyse genetic, morphologic, and acoustic divergence between these lineages that live in the same caves in French Guiana.

**Results:**

A multiplex of 20 polymorphic microsatellite markers was developed using the 454-pyrosequencing technique to investigate for the first time the extent of reproductive isolation between the two lineages and the population genetic structure within lineages. We genotyped 748 individuals sampled between 2010 and 2015 at the 20 nuclear microsatellite loci and sequenced a portion of the cytochrome c oxydase I gene in a subset of these. Two distinct, non-overlapping haplogroups corresponding to cryptic species *P. alitonus* and *P. rubiginosus* were revealed, in accordance with previous findings. No spatial genetic structure between caves was detected for both species. Hybridization appeared to be quite limited (0.1–4%) using microsatellite markers whereas introgression was more common (7.5%) and asymmetric for mitochondrial DNA (mtDNA).

**Conclusions:**

The extremely low rate of hybridization could be explained by differences in life cycle phenology between species as well as morphological and acoustical distinction between sexes in one or the other species. Taken together, these results add to our growing understanding of the nature of species boundaries in *Pteronotus parnelli*, but deserve more in-depth studies to understand the evolutionary processes underlying asymmetric mtDNA introgression in this group of cryptic species.

**Electronic supplementary material:**

The online version of this article (10.1186/s12862-018-1289-8) contains supplementary material, which is available to authorized users.

## Background

Much natural diversity is morphologically hidden [1]. The detection of cryptic species, i.e., genetically divergent species previously classified as a single species due to morphological similarity [1, 2], has significantly increased for all major terrestrial and aquatic taxonomic groups and across biogeographical regions [2, 3] through the use of large-scale DNA sequencing approaches such as DNA barcoding [4, 5]. The discovery of this cryptic diversity has had profound implications for both evolutionary theory and future conservation decisions (see [6–8] for an example involving bats), especially in threatened ecosystems for which biodiversity has likely been underestimated. Morphologically similar species can indeed vary in geographic distribution and ecological requirements, and thus the conservation status among cryptic species belonging to the same species complex can differ (see [9] for an example in Hanuman langur *Semnopithecus entellus*).

Although these cryptic species can usually be identified due to mitochondrial DNA (mtDNA) differences, reproductive isolation is only scarcely investigated. There is indeed an implicit assumption that the molecular genetic divergence among these cryptic species can be taken as a surrogate for reproductive isolation [10–14]. While substantial mtDNA differences between similar or identical-looking individuals can reveal cryptic species, caution is needed as it does not tell us whether the two (or more) cryptic species are truly separate species (see [15] for an illustration of the danger of using only a limited amount of DNA to draw conclusions about evolutionary history). It may instead provide only a glimpse into the evolutionary past of animals that are now one population of interbreeding individuals. To confirm their reproductive isolation, it is necessary to use nuclear markers, which, unlike mtDNA, are inherited from both parents. This allows the question of whether interbreeding occurs or not between the cryptic species to be answered. Another important related question is then how truly cryptic species maintain species cohesion in an area of sympatry. Subtle isolating mechanisms, such as ecologic or recognition systems (e.g. [16–19]), may have evolved, permitting the co-existence of sympatrically living cryptic species. An example of this is *Drosophila paulistorum* for which semi-species are morphologically similar, but have different courtship song patterns [20].

Cryptic species are fairly common in echolocating bats [21]. One of the first major discoveries was that the most widespread European bat species, the common pipistrelle (*Pipistrellus pipistrellus*) had [22] in fact been hiding two cryptic species: *P. pipistrellus* and the soprano pipistrelle (*P. pygmaeus*). Despite being morphologically similar marked divergence in ecological requirements has been evidenced (e.g. [23]). The neotropical insectivorous bat *Pteronotus parnellii* (family Mormoopidae, subgenus *Phyllodia*) also comprises a particularly remarkable example of hidden diversity. Currently, nine cryptic species widespread in Middle America and the Caribbean [24–27] have been recognized within the *P. parnellii* complex. Phylogenetic analyses of mitochondrial genomes suggest that a split occurred in their maternal lineages ∼1.1–2.8 millions years ago, while the entire complex would have shared a common ancestor ∼2.5–6.1 millions years ago [24].

In French Guiana, the presence of two sympatric groups referred to as *Pteronotus* sp3 and sp4 has been proposed (sensu [24, 27]) using several tools: genetics – using mitochondrial molecular markers such as the cytochrome oxidase 1 (CO1), the cytochrome *b* (Cyt *b*), or the y-linked *Dby* genes [24, 25, 28] –, morphology [24, 28], and bioacoustics [28, 29]. Recently these two groups have been diagnosed as two distinct species, *P. rubiginosus* corresponding to *P*. sp4 [27] while *P*. sp3 has been named *P. Alitonus* [27]. However, these studies were limited by the number of samples when genetic and acoustic or morphological data were gathered on the same individuals, or because genetic and other data were collected on different individuals. Morphological studies failed to provide diagnostic characters to distinguish between members of this complex, but differences occur in their echolocation call frequency [28, 29]: *P. rubiginosus* uses a peak echolocation frequency of 53 kHz, *P. Alitonus* 59 kHz. It is evident that we still know very little about these newly discovered groups in terms of their preferred habitats, prey and roosting places, their breeding ecology or their population structure and potential for interbreeding - i.e., not enough information has been gathered as yet to formally describe them as new species.

In this study we have investigated colonies of *Pteronotus* sp. roosting in four caves in French Guiana during the period 2010 to 2015. We combined different approaches in order to investigate genetic, morphological and acoustic divergence between the two groups. For the first time we tested whether the identified groups in the caves interbreed using autosomal microsatellite markers and searching for hybrids in the sampled populations. Lastly, we explored the spatial genetic structure of *Pteronotus* sp., notably whether caves affect the genetic structure of the population of *Pteronotus* sp. For the DNA studies we used the 5′ half of the mitochondrial CO1 and developed a panel of 20 specific microsatellites in one multiplex using the 454-pyrosequencing technique. The study’s aim was to make comprehensive genetic comparisons that would reveal relationships between the two groups and to understand their microevolutionary history - which might include hybridization and introgression patterns.

## Results

### Molecular analysis

#### Species identification

**Fig. 1:**
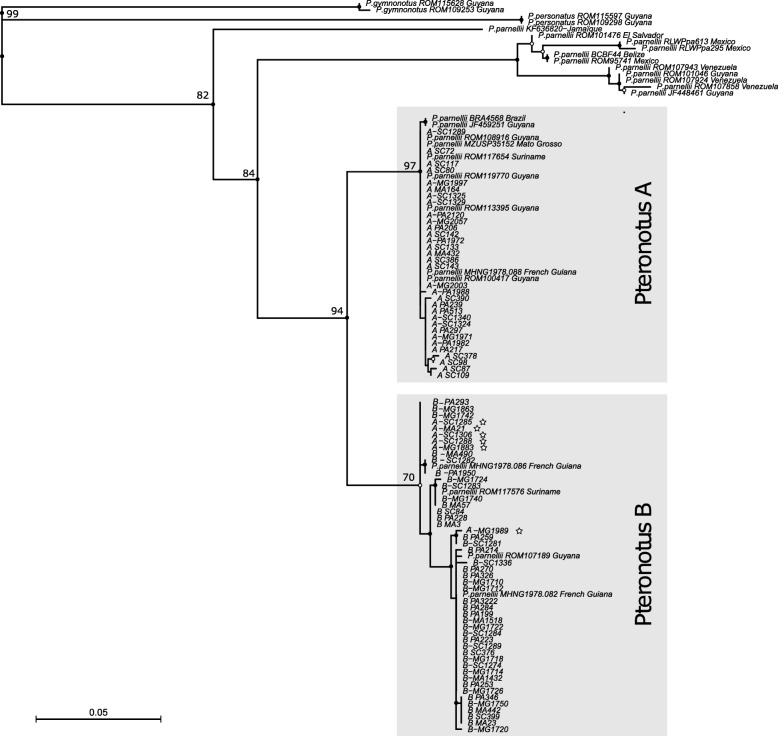
Phylogenetic relationships among 26 external COI sequences of *Pteronotus* and 80 COI sequences of *Pteronotus* from our study. Specimen references of the 26 external sequences are listed in Additional file 1: Table S1. Stars represent hybrids (*n* = 6, identified as *Pteronotus* A with nuclear DNA while having mtDNA from *Pteronotus* B). In sample names, the first letter (“A” or “B”) refers to the nuclear assignation of individuals to one species or the other while grey squares refer to the mtDNA assignation

The phylogenetic reconstruction (Fig. 1, Additional file 1: Table S1) showed that the 80 *Pteronotus* individuals sampled in the present study display two highly supported groups, corresponding to *P. alitonus* (hereafter *Pteronotus* B) and *P. rubiginosus* (hereafter *Pteronotus* A) previously evidenced by [24, 27, 28].

Divergence of COI sequences was very small within the A and B groups (0.15 and 0.77% on average, respectively) but much larger between the groups (5.72%).

#### Nuclear genetic analysis

We built a multiplex of 20 microsatellite markers (Additional file 1: Table S2). These 20 microsatellite markers were successfully amplified for 748 bat samples with a mean amplification rate of 98.7%. All markers were polymorphic, with the number of alleles ranging between 2 and 17.

Consistently with the phylogenetic reconstruction, the STRUCTURE analysis over 20 microsatellites combined with Evanno’s method detected two sharply differentiated clusters (Additional file 1: Table S3). Among the 748 genotyped individuals, 325 belonged to *Pteronotus* A while 423 individuals belonged to *Pteronotus* B (see Table 1). The allelic diversity at the 20 microsatellite loci was comparable in both groups, but not identical (Table 2). The two groups were both present in all four caves sampled.

**Table 1:** Number of *Pteronotus* A and B genotyped at each field session

Year	Month	*Pteronotus A*			n(A)	*Pteronotus B*			n(B)	n
		F	M	NS		F	M	NS		
2010	August	0	1	0	1	3	4	0	7	8
2010	September	24	97	0	121	29	70	2	101	222
2011	July	7	63	0	70	26	37	0	63	133
2012	July	17	32	0	49	48	26	0	74	123
2015	October	24	54	6	84	71	105	2	178	262
Total		72	247	6	325	177	242	4	423	748

*F* Females, *M* Males, *NS* Not sexed, *n(A)* number of *Pteronotus* A, *n(B)* number of *Pteronotus* B, *n* total number of *Pteronotus*

**Table 2:** Characteristics of the twenty microsatellite markers developed for *Pteronotus parnellii*

Locus	*Fst*	*Pteronotus* A				*Pteronotus* B			
		*Fis*	*Na*	*Ho*	*He*	*Fis*	*Na*	*Ho*	*He*
PP01	0.23	−0.095	4	0.465	0.424	0.049	7	0.730	0.768
PP02	0.17	−0.007	4	0.477	0.473	0.021	4	0.280	0.285
PP03	0.18	−0.070	3	0.465	0.434	−0.030	4	0.752	0.729
PP05	0.11	0.068	4	0.474	0.508	−0.006	5	0.711	0.706
PP08*	0.41	0.061	3	0.315	0.335	0.330	8	0.513	0.764
PP09	0.15	0.012	5	0.615	0.622	0.020	8	0.834	0.851
PP10^μ^	0.42	0.310	11	0.570	0.821	−0.002	11	0.839	0.837
PP12	0.09	0.004	3	0.108	0.108	0.062	4	0.374	0.398
PP13	0.13	NA	1	0.000	0.000	−0.038	2	0.260	0.250
PP14	0.16	0.044	10	0.674	0.704	−0.010	11	0.740	0.732
PP15	0.24	−0.043	9	0.834	0.798	0.011	8	0.707	0.714
PP16^μ^	0.19	0.110	4	0.482	0.539	0.003	8	0.861	0.862
PP17	0.15	−0.010	7	0.738	0.730	0.014	9	0.757	0.767
PP18	0.07	0.004	13	0.791	0.793	−0.022	17	0.856	0.837
PP21	0.09	0.030	4	0.517	0.532	0.013	8	0.770	0.779
PP22^#^	0.08	−0.008	5	0.680	0.674	0.017	5	0.530	0.538
PP23	0.08	−0.024	4	0.683	0.666	−0.015	6	0.751	0.740
PP24^#^	0.61	−0.043	6	0.441	0.422	0.211	4	0.186	0.236
PP26	0.06	0.020	5	0.397	0.404	0.004	6	0.621	0.622
PP27	0.01	0.009	4	0.443	0.446	−0.003	3	0.331	0.329

*Fst* and *Fis* values correspond to Weir and Cockerham’s estimates. The number of alleles is given in the *Na* column and the observed and expected heterozygosity are provided in the *Ho* and *He* columns, respectively. Loci with a star (*), a mu (μ) or a sharp (#) correspond to loci with significant null alleles frequency, loci in Hardy-Weinberg disequilibrium in species A or B, respectively. All these loci were excluded in population genetics analysis

One locus showed significant signs of null allele frequency (PP08, *p* = 0.04) and four others appeared to be in Hardy-Weinberg disequilibrium for at least one of the two identified species (PP10 and PP16 for *Pteronotus* A, PP22 and PP24 for *Pteronotus* B, *p* < 10^− 4^). We did not find any loci in linkage disequilibrium. Consequently, we ran STRUCTURE using 15 loci to redefine the two clusters, and considered these 15 markers in subsequent analyses. The reduction of the number of markers did not change the assignation of individuals to cluster A and B. Over these 15 microsatellite markers, the two species presented similar mean number of alleles per locus: 6.71 for *Pteronotus* A and 7.82 for *Pteronotus* B.

The two *Pteronotus* species were significantly differentiated with a *F*_*ST*_ value of 0.139 [0.104–0.171]_95%_. Within species, only *Pteronotus* A males displayed significant genetic differentiation (global *F*_*ST*_ values: 0.002, *p* = 0.007) and structure between caves (only significant between PA and SC caves; see pairwise *F*_*ST*_ values, Additional file 1: Table S4). Additionally, we did not find any significant sex-biased dispersal (*p* > 0.05).

#### Hybrid detection

Two thresholds, TP1 and TP2, were determined to identify hybrids based on the 15 microsatellite markers. They corresponded to the lowest *q*-value reached by simulated parents in each cluster. For each genotyped individual, either the mean *q*-value (conservative approach) or the lower bound (relaxed approach) of the credibility interval returned by STRUCTURE was compared to those thresholds to determine whether the individual was hybrid. The simulation procedure led us to consider 0.90 and 0.76 as values for TP1 and TP2. Accordingly, one putative hybrid was detected with the “conservative” approach while 30 putative hybrids were detected with the “relaxed” approach. A further simulation study revealed that the probability to identify parental individuals as hybrids, either with the conservative or relaxed approach, was null with these thresholds used in a configuration where the number of hybrids is lower than the number of parental forms. No hybrid was detected using the computer program NEWHYBRIDS.

Among individuals for which we have sequenced mitochondrial DNA (*n* = 80), all 42 individuals assigned to *Pteronotus* B with nuclear DNA had *Pteronotus* B mitochondrial sequences. In contrast, 6 individuals out of the 38 assigned to *Pteronotus* A with nuclear DNA were found to have *Pteronotus* B mitochondrial sequences (see Fig. 1).

### Morphology

**Fig. 2:**
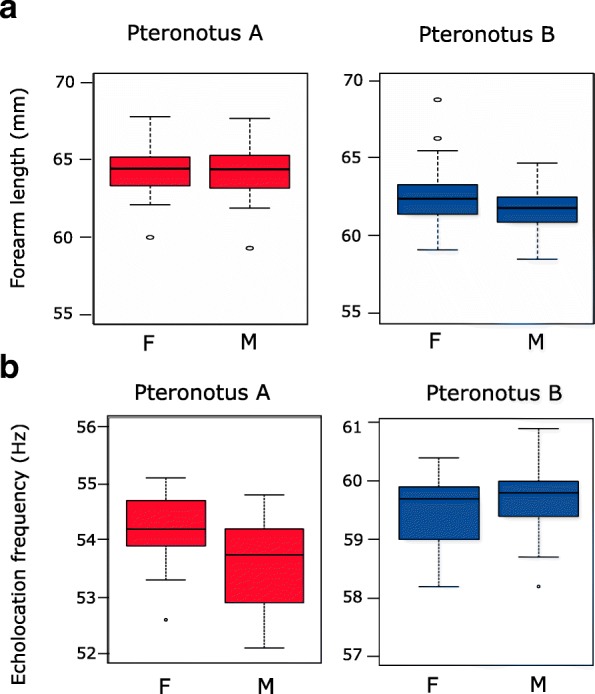
**a** Forearm length, and **b** Echolocation frequency in females (F) and males (M) depending on the group

Only 23 of the 30 individuals identified as putative nuclear hybrids with the relaxed approach have morphometric data. Excluding these 23 individuals to be conservative as well as the 6 mtDNA hybrids (see above), *Pteronotus* A (*n* = 166) had a mean forearm length of 64.37 (95% CI [64.13;64.56]) vs 62.06 (95% CI [61.90;62.22]) for *Pteronotus* B (*n* = 300) (Fig. 2a). This difference between both groups was found significant even after correcting for the potential confounding effect of sex (F = 293.17, df = (1462), *p* < 10^− 16^).

The global comparison between sexes showed non-homogeneous forearm length in at least one group (F = 8.38, df = (2462), *p* = 2.7 × 10^− 4^). The post-hoc analysis revealed that the effect of sex was significantly different between the two groups (F = 5.90, df = (1462), *p* = 1.5 × 10^− 2^) and was only found significant in group B (*t* = 4.07, df = 298, *p* = 6 × 10^− 5^, forearms being longer in females; in group A: *t* = − 0.38, df = 164, *p* = 0.73).

Due to the absence of F1 and to a small number of morphological data for putative hybrids (Additional file 1: Figure S5), we could not compare hybrid and parental morphological features.

### Echolocation

Among the 262 (73 *Pteronotus* A, 171 *Pteronotus* B, 13 putative nuclear hybrids identified with the relaxed approach and 5 mtDNA hybrids) microphone recorded individuals, 146 (of which 15 are hybrids) were precisely measured for the Frequency of Maximal Energy (FME) with BatSound (FME dataset). Excluding the 15 hybrid individuals of the FME dataset and the four outliers from group B (pure individuals), *Pteronotus* A (*n* = 60) emitted at a call frequency situated around 53 kHz (53.73 kHz, 95% CI [53.51;53.96]) while *Pteronotus* B (*n* = 67) emitted at a call frequency situated around 59 kHz (59.64 kHz, 95% CI [59.49; 59.78]) (Fig. 2b). This difference between both groups was found significant even after correcting for the potential confounding effect of sex (F = 2080.8, df = (1116), *p* < 10^− 16^).

The global comparison between sexes showed non-homogeneous echolocation frequency in at least one group (F = 6.32, df = (2116), *p* = 2.4 × 10^− 3^). The post-hoc analysis revealed that the effect of sex was significantly different between the two groups (F = 10.68, df = (1116), *p* = 1.4 × 10^− 3^) and was only found significant in group A (*t* = 2.92, df = 53, *p* = 5.2 × 10^− 3^, frequencies being higher in females; in group B: *t* = − 1.37, df = 63, *p* = 0.18).

Again, due to the absence of F1 and to a small number of acoustic data for putative hybrids (see Additional file 1: Figure S5), we could not compare hybrid and parental acoustic features (see Additional file 1: Figure S5).

### Reproductive periods

Regarding *Pteronotus* A, only two females (over 24) were found to be pregnant in July with one in MA and one in PA, and one lactating female was found in October (over 24). No juveniles were found during the different field periods. Concerning *Pteronotus* B, pregnant (27 over 74) and lactating (15 over 74) females were found in July and post-lactating (33 over 103) females between late August and October in the MA and PA caves (Additional file 1: Table S6). Ten juveniles were captured in September in the PA cave.

## Discussion

In this study, molecular, acoustic, and morphological differentiation has been analyzed in *Pteronotus* sp. collected from four caves in French Guiana. Using COI - a mtDNA marker commonly employed for species barcoding - our phylogenetic reconstruction of *Pteronotus* reveals two distinct, non-overlapping haplogroups corresponding to cryptic species *P. alitonus* and *P. rubiginosus*, in accordance with previous findings (see [24, 25, 27, 28]). The smallest divergence between *P*. *alitonus* and *P.* *rubiginosus* is still almost 3 times larger (2.7) than the largest within group divergence – a typical value for interspecific differences in mammals [5, 30]. We found good agreement between mitochondrial and nuclear microsatellite markers, i.e. the two mtDNA lineages encompass two distinct nDNA genetic clusters. The *F*_*st*_ value of 0.139 between *P. alitonus* and *P. rubiginosus* indicates a strong population genetic differentiation in the 15 microsatellite markers used.

### Reproductive season

*P. alitonus* and *P. rubiginosus* appear to have an overlapping reproductive season. However the very low proportions of pregnant and lactating *P. rubiginosus* females compared to *P. alitonus*, together with the absence of juveniles, suggest a small shift in the reproductive season between the two species. Alternatively, we cannot exclude the possibility that *P. rubiginosus* uses breeding sites other than caves (e.g., buildings, underside of bridges), potentially reducing the competition with *P. alitonus* for food resources. The identification of a colony with pregnant (9 over 24 females) and lactating (14 over 24 females) females of *P. rubiginosus* in the roof of a village house in June 2017 may confort this second hypothesis. It was also suggested that *P. alitonus* prefers to forage in more dense forest than *P. rubiginosus* [27]. Such spatial separation of foraging habitats may reduce competition between the two species thereby explaining their co-existence in the same area.

### Phenotypic differentiation

When comparing forearm length between the two cryptic species, we found a broad overlap between measurements showing that the species distinctions did not precisely match the genetic differences (see also [24, 28, 31]). We also showed that *P. rubiginosus* is slightly larger than *P*. *alitonus*, as previously reported by de Thoisy et al. [28] in the same area, in López-Baucells et al. [31] in a diversity of habitats in the Central Brazilian Amazon. Interestingly, Pavan et al. [27] showed that this phenotypic difference is enhanced in areas where the two species occur in sympatry – a result which is expected in species undergoing character displacement [27, 32]. Furthermore, in our study we observed that females have a slightly larger forearm length than males in *P. alitonus.* Sexual dimorphism had already been demonstrated by Clare et al. [24] in skull size, with males having larger ones. Such subtle size differences between males and females suggest that *P. alitonus* may vary in sexual selection intensity over its distribution area – as it was observed for *Rhinolophus ferrumequinum* ([33]; see also [34]). However, such dimorphism may be closely linked with habitat types and thus with natural selection rather than sexual selection. For example, the existence of habitat-specific sexual dimorphism has been reported in *Anolis* lizards [35]. Thus, females of *P. alitonus* may have evolved larger forearms than males because it may confer reproductive advantages [34, 36] depending on their environmental conditions (e.g., food availability, competitors).

As previously reported for French Guiana and in the Central Amazon [24, 28, 29, 31], the two cryptic species correspond to two entirely distinct phonic types – one displaying frequencies of maximum energy around 53 kHz (*P. rubiginosus*), and the other around 59 kHz (*P. alitonus*). Jiang et al. [37] and Lin et al. [38] suggested that variations of 5–7 kHz do not impact the ability to detect prey and thus, should not affect resource use. The two species occupy the same caves, which are a limiting resource in the studied area, suggesting that they coexist without major ecological competition either for roost caves or for prey, as already discussed for other cryptic species such as *Rhinolophus mehelyi* and *R. euryale* [39]. We thus hypothesize that the different acoustic calls may have evolved to facilitate intraspecific and interspecific communication/recognition, rather than to facilitate resource partitioning. Though unlikely [40], a neutral explanation for this observation cannot entirely be ruled out.

Furthermore, *P. rubiginosus* - which have longer forearms - use lower peak frequency of echolocation calls (53 kHz) than *P. alitonus* (59 kHz), in accordance with the mass-signal frequency allometry (e.g. [41, 42]). However, this size-dependent effect in call frequencies cannot explain the inter-sex variation observed in *Pteronotus* sp. While *P. alitonus* females have slightly larger forearm length than males both genders emit at the same call frequency. On the contrary, although *P. rubiginosus* bats do not exhibit dimorphism in forearm length between the sexes, we did find that females emit at higher frequencies than males. By contrast with *P. alitonus*, sexual selection may act more on echolocation call frequencies in males than body size in *P. rubiginosus*. Such variation in call frequencies between the sexes has not been reported in previous studies on *Pteronotus* species [24, 28]; however, the number of individuals sampled was limited and no information about the sampling period was given. Although we have no idea if males were active or not during the sampling periods for both species (handling animals giving rise to fluctuating testis size we had no reliable indicator), we suggest that certain bat species - at least *P. rubiginosus* - can change their call frequency during the mating period. For example, Grilliot et al. [43] have observed in *Eptesicus fuscus* that bats can modulate their call according to functional context, making them monomorphic for activities such as foraging, but dimorphic during mating activity. Acoustics may thus play an important role in sex recognition in *Pteronotus* and/or indicate some aspects of male condition or quality at least in *P. rubiginosus* (as highlighted in *R. mehelyi* [44]). Such differences in calls may limit mating between species in *Pteronotus*. Some studies have suggested that changes in echolocation frequency (for instance *Rhinolophus philippinensis*) are associated with assortative mating, and ultimately reproductive isolation and speciation, regardless of external barriers to gene flow [18].

### Hybridization

We found evidence of limited hybridization between the two species using the 15 microsatellite markers: only 1 individual (conservative approach) and 30 individuals (relaxed approach) out of 748 showed signs of hybridization. On the long term however, such events leave traces that are best observed in the mitochondrial genome: 6 individuals out of 80 contain COI sequence of the other species. The most striking finding is that in all cases mtDNA introgression has occurred asymmetrically, from *P. alitonus* to *P. rubiginosus* (6 out of 38 *P. rubiginosus* have mtDNA from *P. alitonus* while all 42 *P. alitonus* contain the mtDNA of *P. alitonus*). Our proportion of mtDNA hybrids in *P. rubiginosus* (0.158) is higher than that previously reported by Clare et al. [24] who identified - in the lowlands of the Guyana Shield - only one hybrid out of 61 specimens (35 *P. alitonus* and 26 *P. rubiginosus*), but similarly, this hybrid recovered the mitochondrial DNA of *P*. *alitonus* (giving a proportion of hybrids of 0.04). Similar results have been reported in bats between the two sibling bat species *Myotis myotis* and *Myotis blythii* [45], and *Rhinolophus sinicus septentrionalis* and *R. s. sinicus* [46]. Many different scenarios have been suggested to explain biased hybridization in mtDNA such as sex-biased dispersal, asymmetry in mate choice, differential production of offspring, demographic dynamics of local and colonizing species, differential selection, or a combination of these effects [47]. Differentiating between the possible causes is complex but some of them can be tentatively discussed to explain this unexpected asymmetry of mtDNA lineages between *P. alitonus* and *P. rubiginosus*.

Based on our analysis of genetic data at the scale of the colony, sex-biased dispersal is an unlikely explanation for the observed asymmetry since there is no evidence that males and females - of both species - disperse at different distances.

The observed unidirectional introgression of mtDNA could alternatively reflect a propensity of mating between *P. rubiginosus* males and *P. alitonus* females but not the opposite (*P. rubiginosus* females and *P. alitonus* males). Such asymmetry in mating between the sexes has been observed in insects [48] and in birds [49] but to our knowledge very few data regarding mating behavior are available for bats (see, e.g., [50–52]) and no data exist in particular for our two species. For example Bogdanowicz et al. [53] proposed that swarming behavior at swarming sites, where high number of bats belonging to several species meet, is an important factor that could explain hybridization among the three bat species they studied. Considering that both species coexist in the same habitats, form mixed maternity colonies in the caves, that their reproductive season seems to overlap, that they differ in call frequency, as well as the extremely low rate of hybridization on microsatellite markers, we may consider asymmetry in mating between the sexes as anecdotal. Phenotypic differences between *P. alitonus* and *P. rubiginosus*, both in size and regarding the presence or absence of sexual dimorphism (see also [24, 27, 28]), could help species in the recognition of conspecifics and participate in the limitation of hybridization. As already mentioned, recognition between sexual partners can also be achieved through acoustic cues such as echolocation. This acoustic dimorphism may also help avoiding hybridization by erroneous recognition of conspecifics.

Finally, the asymmetry of introgression could also arise if the mtDNA of *P. alitonus* has some selective advantage that promotes its introgression in an alternative environment (a scenario proposed to explain the spread of mtDNA in some hare populations, [54] or in a foreign genetic background [55]. The mitochondrial genome plays a central role in cellular energy provision and can affect different life-history traits like life span and fertility, and/or behavior such as activity and exploration (e.g. [56]). An increasing number of studies have indicated that mtDNA appears to be under selection (e.g. [57–59]) but arguments in favor of this hypothesis are, as yet, far from being established for *Pteronotus*. That asymmetric hybridization of mtDNA occurs at a large geographical scale (Guyana Shield) but at different frequencies, can however give some support to this hypothesis.

### Genetic structuring of populations

For the first time, these two cryptic species have been studied using autosomal microsatellites. These new markers also allowed us to shed light on the genetic patterning of the populations. In our dataset, the level of genetic differentiation is an order of magnitude higher between species than within species (*F*_ST_ = 0.13 versus *F*_ST_ = 0.002, respectively), suggesting the existence of high gene flow between caves for the two species. Thus the cave does not appear to be a structuring unit as the populations of *P. alitonus* and *P. rubiginosus* seem to form two unique populations covering distances up to 80 km (from the Nouragues region to the Kaw Mountains). One possible explanation for this lack of genetic structure is that the distance between caves is lower than the dispersal distance of both species. Thus, the structuring unit may be an ensemble of caves rather than each cave.

## Conclusions

In agreement with previous studies, our genetic, morphological, and acoustic results justify the existing classification of *Pteronotus* into two species - *P. alitonus and P. rubiginosus* - in French Guiana. Much work remains to be done to increase our knowledge of the evolutionary mechanisms that generate the process of speciation in the *Pteronotus* complex and to identify the causes of incongruence between mitochondrial and nuclear data. Furthermore, it remains to determine whether call differences have resulted from food competition or from intraspecific recognition in the context of sexual selection. Investigation of foraging behavior and diet of each species is crucial to analyze whether call frequency differences between the two species influence habitat or prey preference. We also need to identify the reproductive period of both species as well as their reproductive sites, and in particular whether *P. alitonus* and *P. rubiginosus* are both able to modulate their echolocation call during the mating period. The presence of a sexual dimorphism in echolocation in *P. rubiginosus* but not in *P. alitonus* could indeed reflect a slight shift in their breeding phenology, thereby limiting current genetic interaction between the two species. Future studies should also look deeper into the relationship between morphological, call frequencies and habitat use by sympatric and allopatric populations of *Pteronotus* species.

## Methods

### Study area and bat capture

**Fig. 3:**
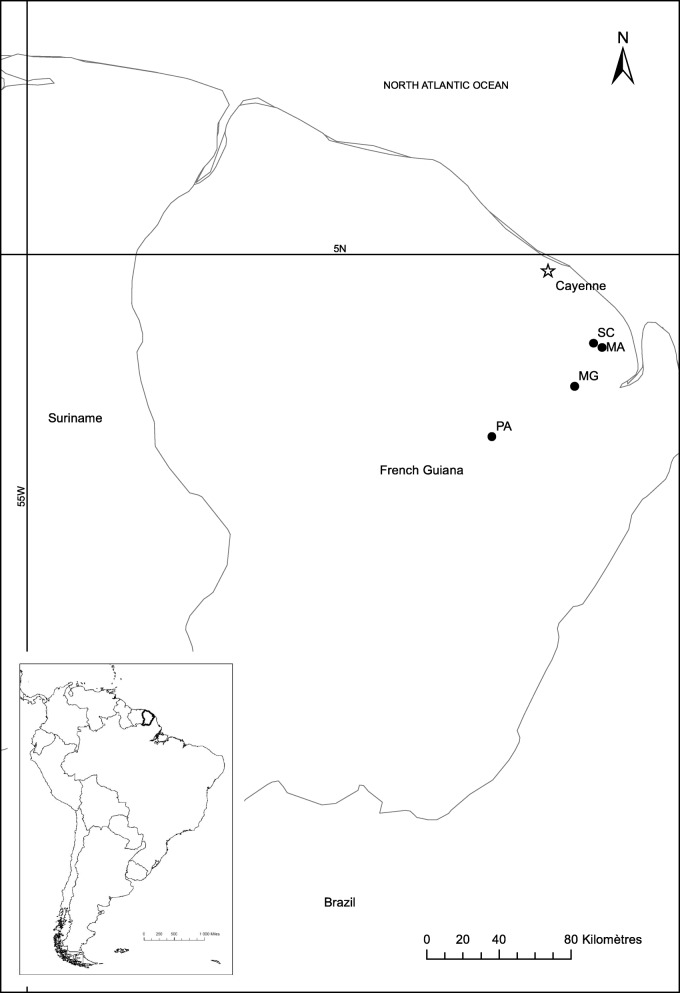
Location of the sampled sites in French Guiana. MA: Mathilde cave, SC: Scierie cave, PA: Parfums cave, MG: Montagne des gouffres cave

*Pteronotus* bats were sampled from 4 caves in the tropical rainforests of French Guiana (Fig. 3): Mathilde (MA), Scierie (SC), Parfums (PA) and Montagne des gouffres (MG). There were four sampling sessions: late August/September 2010, July 2011, July 2012, October 2015, except for MG which was only sampled in October 2015. Distances between caves vary from 12 to 15 km between MA and SC to 80 km between MA or SC and PA. MG is 55 km from PA and 25 km from SC and MA.

Bats were caught in a Two-Bank HarpTrap (AUSTBAT Research Equipment, Victoria, Australia; catching surface of 4.0 m^2^) as they left the caves for foraging. We placed them individually in cotton bags until sampling. Adults were distinguished from juveniles by trans-illumination of the cartilaginous epiphyseal plates in the phalanges [60]. We estimated the reproductive status of each bat by examining the development of testes and caudae epipidymides for males, and the development of mammary glands and of nipples for females. Pregnant females were identified through palpation. Lactation was confirmed by gentle squeezing the mammary glands and nipples. Non-juvenile individuals were classified as inactive or active males, and inactive, pregnant or lactating females. We measured forearm length (with a dial caliper) and body weight (with an electronic scale) to the nearest 0.05 mm and 0.1 g, respectively. Tissue sample for DNA analysis was collected from the wing membrane (patagium) using a 3-mm diameter biopsy punch (Kai Industries, Gifu, Japan) and preserved in 70% ethanol solution until DNA extraction. Bats were released at the place of capture after sampling.

### Molecular analysis

#### Identification of microsatellites via high-throughput sequencing

Ten individuals were randomly chosen in order to build a pool of DNA. This pool was then used to design microsatellite markers based on the GS-FLX® method [61]. This method consists of firstly, a fragmentation of the genomic DNA. The method then proceeds by an enrichment of microsatellite sequences through the addition of primers with common STR patterns (TG, AAC, AAG, ACAT, TC, AAG, ACG, ACTC). Finally, the enriched DNA is amplified with a High Fidelity Taq polymerase. The bands thus obtained were quantified in order to have a minimum of 5 ng of genomic DNA. The resulting sequences were analyzed with QDD software [62].

#### Multiplex building and amplification

Thirty microsatellite markers were chosen among the set of 3487 markers identified via GS-FLX®. A Simplex Step was performed in order to verify their proper amplification. More steps of primer design and primer concentration adjustment followed in order to build a multiplex of 20 microsatellite markers. During this step of multiplex building, tetranucleotides were privileged because we expected them to have a lower mutation rate - although this is uncertain given the high interspecies variability [63, 64] - and because of their higher legibility in subsequent computer analyses.

#### Sequencing and genotyping

A total of 748 *Pteronotus* specimens out of the 1349 captured were genotyped at 20 microsatellite markers (see Additional file 2: Table S7). Total genomic DNA was extracted using purification column kits (Nucleospin 96 Tissue, Macherey-Nagel) following the manufacturer’s instructions and in the presence of positive and negative controls. PCR reactions were carried out in 96-well microplates with three negatives and positive amplification controls to verify lack of contamination, in a total volume of 10 μl (2 ng/μl of DNA, 1.58 μl of a mix of primers at a concentration between 0.06 and 0.5 μM in the final PCR, 5 μl of 2X Mastermix). The samples were first denatured at 95 °C for 5 min. Then, 30 cycles followed (denaturation at 95 °C for 30s; hybridization at 58 °C for 90s; elongation at 72 °C for 30s) and a final elongation step at 60 °C for 30 min. PCR products were resolved on a ABI PRISM 3130XL capillary sequencer (Applied Biosystems) with formamide (denaturing conditions) and an internal size marker (600 liz; Applied Biosystems) in one migration. The electrophorograms were analyzed using GENEMAPPER 4.1 (Applied Biosystems/Life Technologies) independently twice by different operators. Results were then compared, and ambiguous loci were set to missing data.

We also amplified the cytochrome c oxydase I (COI) mitochondrial marker (500 bp) for 80 individuals randomly chosen using the primers Bat-COI-01-F (5’-TGAGCAGGAATAGTAGGCAC-3′) and Bat-COI-03-R (5’-CGGCAGGGTCAAAGAATGTG-3′). Sequencing reactions were performed with the corresponding amplifying primers using a BigDye Terminator Cycle Sequencing Kit v.2.0 (Applied Biosystems, USA) and run on an ABI 3730 automated sequencer (Applied Biosystems, USA).

#### Species identification and hybridization detection

In order to identify the species to which the sampled specimens belonged, we built a tree based on the COI sequences of these 80 individuals and added 28 samples obtained in previous studies [24, 25, 28] for which COI sequences were retrieved from Genebank (see Additional file 1: Table S1 for the detailed list of GenBank sequences used). *Pteronotus gymnonotus* and *P. personatus* were used as outgroups.

COI sequences for the 108 specimens (80 + 28) were aligned using MACSE [65] with default parameters. The alignment was trimmed with trimAl v1.2rev59 [66] using the -*gappyout* option. Phylogenetic reconstruction was achieved using the IQTree Maximum Likelihood method [67] after automatic selection of the best fitting model (X + F + G4) and 1000 ultrafast bootstrap replicates were performed to get support values associated with the branches.

The genetic distance between COI sequences was also computed for all pairs of sequences in order to compare within and between COI divergence. This was done in R language [68] using the *dist.dna* function of the “ape” package v.5 [69] with the “raw” method (simple computation of the proportion of sites that differ between each pair of sequence).

The group identification of all individuals was also carried out using the microsatellites we developed specifically for *Pteronotus*, along with the detection of hybrids, using the Bayesian software STRUCTURE 2.3.4 [70, 71]. We used the admixture model with correlated allele frequencies between populations without any prior information on the putative group affiliation of individuals in all analyses. The program was run with a Monte-Carlo Markov chain length of 1.000,000 after a burn-in of 300,000 iterations. For the identification of groups, we first validated the number of clusters that best described the data following the method of Evanno et al. [72] implemented in STRUCTURE HARVESTER online web 0.6.94 [73]. For this purpose, we ran STRUCTURE for values of *K*, the number of clusters, ranging from one to four, twenty independent runs being carried out for each value of *K.* The outputs from the 20 runs corresponding to the optimal value of *K* were then compiled using CLUMPP [74] to access consensus individual probabilities of assignment (*q-*values), and their 90% credibility interval, to the different clusters identified.

For the detection of hybrids, we selected the 80 individuals presenting the highest *q-*values in each cluster and used them to simulate individuals belonging to different hybrid classes (300 simulated individuals from parental classes, F1, F2, first generation backcrosses) using the function *hybridize* from the package *adegenet* [75] implemented in R [76]. These individuals were then pooled together and analysed using STRUCTURE to obtain their *q-*values for the *K* clusters identified in the real population. Based on these *q-*values, we defined two thresholds (two thresholds because *K* = 2, see Results section) corresponding to the lowest *q-*value reached by a simulated parent in each cluster (TP1 and TP2). These thresholds were then used to categorize the sampled individuals either as a parent or a hybrid either on the basis on their mean *q-*value (a conservative approach that detects individuals that are clearly hybrids) or the lower bound of their 90% credibility interval (a relaxed approach that detects all individuals presenting cues of hybridization as hybrids). We ran additional simulations in order to assess whether hybrids detected with the relaxed approach may be misclassified parental forms. To do so, we simulated again hybrid classes (300 individuals from each parental population, 10 F1, 15 backcrosses to each population) using the function *hybridize* and analysed the simulated individuals with STRUCTURE. We repeated the simulations thirty times and counted the number of parental forms detected as hybrids with the methods previously described. For this additional study, we simulated a few hybrids (10 F1, 15 backcrosses to each parental form) to consider a scenario consistent with the proportion of hybrids detected in the sampled population. We used the relaxed approach to select unambiguously parental groups for the morphologic and acoustic analyses. The detection of hybrids was also performed using the computer program NEWHYBRIDS 1.1 [77] This analysis was carried out using Jeffreys’ prior with a burn-in period of 50,000 and the MCMC chain of 100,000 iterations.

#### Genetic variability and population genetics of Pteronotus sp.

Once the group of each individual was determined, microsatellite loci were examined for null (non-amplifying) alleles in each species using the program Micro-checker v.2.2.3 [78]. The significance of null allele frequencies was assessed using a binomial exact test following De Mêeus et al. [79]. We also tested for deviation from Hardy-Weinberg equilibrium and linkage disequilibrium for each pair of loci with FSTAT 2.9.3.2 [80]. *P*-values were adjusted using a Bonferroni correction. All subsequent analyses were performed without the loci for which we detected significant signs of null allele frequencies or deviance from Hardy-Weinberg equilibrium.

Allelic richness and Weir and Cockerham’s *F*_*ST*_ estimates for each group were assessed using FSTAT 2.9.3.2 [80]. In order to test the role of the caves in the structuration of the bat populations, we also calculated pairwise *F*_*ST*_ between each pair of caves. Exact G-tests of population differentiation [81] were performed to test for differentiation significance. Finally, sex-biased dispersal tests were achieved based on the *F*_*ST*_ because it is considered to be the most powerful statistic available for this test [82]. A thousand randomizations were made for each species and unilateral tests were performed setting either male or female as the dispersing sex.

### Morphology

Only adults were measured (presented in Additional file 2: Table S7). We performed a two-way ANOVA to determine whether there were differences in forearm (FA) length between sexes and between groups. Because sexual dimorphism may be quite different between species [24, 28], we tested the effect of sex by comparing the nested models Sex*Group and Group using the classical Fisher’s test of linear models. Rejecting the null hypothesis would then reveal the presence of a sexual dimorphism in at least one species. In that case, post-hoc *t*-tests comparing males and females were performed in each group considered independently and the hypothesis that sexual dimorphism is identical in both groups was tested by comparing models Sex*Group and Sex+Group. Recorded body weights were not analysed because of the high variability according to time (before or after being fed) and period of capture (parturition state). Statistical analyses were performed with R version 3.3.3, and significant data were determined as having *p*-values less than 0.05.

### Echolocation frequency

Echolocation calls were recorded in 2015 from 262 *Pteronotus* bats held at 30 cm from a ZoomH2 microphone (Zoom Corporation, Japan) linked to a Pettersson D240X ultrasonic detector (Pettersson Elektronik AB, Uppsala, Sweden) (presented in Additional file 2: Table S7). Signals were analyzed with Bat Sound Pro 3.4 ultrasound analysis software (Pettersson Elektronik AB, Uppsala, Sweden) based on spectrograms with a Hanning window at a sampling frequency of 44.1 kHz and a Fast Fourier transformation (FFT) size of 512. From two to five calls were analyzed for each bat, and for each recorded call, the harmonic containing most energy (Frequency of Maximal Energy: FME) was identified from the power spectrum and measurements taken from the constant frequency (CF) component of the call (see [28, 83]). Among genotyped individuals, a two-way ANOVA was applied to evaluate differences in frequency between groups and sexes. We followed the same procedure as described above for forearm length. For the echolocation variable, five outlying points were identified in *Pteronotus* B (with very low echolocation value compared to the rest of the sample in this group). These outliers were removed from the analysis.

### Reproductive periods

In order to have an inkling as to the reproductive periods of the two groups of *Pteronotus* identified, we analysed the reproductive status for each individual as well as their age (juvenile or adult) during the different sampling periods, without any statistical test due to the low number of individuals sampled for each period.

## Additional files

Additional file 1: **Table S1.** Table. List of external COI sequences used in this study. The sequence in bold corresponds to type-locality of *P. rubiginosus*, caught in MatoGrosso, Brazil compared to *P. sp4* in De Thoisy et al. (2014). **Table S2.** Characteristics of microsatellite markers. **Figure S3.** Graphical representation of assignation probabilities for *Pteronotus* A and *Pteronotus* B sampled. Each individual (x axis) is represented by a vertical bar divided in two parts according to its assignation probability (y axis) in each of the two clusters. **Figure S4.** Graphical representation of the difference between the Conservative and the Relaxed approach for the detection of hybrids. **Table S5.** Pairwise *Fst* values between caves for each species and both sexes. **Table S6.** Life cycle of *Pteronotus* A and B. PRE = Pregnant, LAC = Lactating females, PLAC = Post-lactating females, NS = No status (non pregnant, non-lactating, non post-lactating females, and non breeding males). n(A): number of *Pteronotus A* (number of adult females in parentheses); n(B): number of *Pteronotus B* (number of adult females in parentheses); n: total number of *Pteronotus*. (DOCX 319 kb)

Additional file 2: **Table S7.** Data used in the study, including sex, forearm length, FME, genotype at 20 microsatellite markers for 748 *Pteronotus* sp. Data in bold are individuals identified as hybrids using microsatellite (*N* = 30) or mitochondrial data (*N* = 6). (XLSX 253 kb)

## Abbreviations

°C: Degrees Celsius
CO1: cytochrome oxidase 1
CytB: cytochrome b
DNA: Deoxyribonucleic acid
FME: Frequency of Maximal Energy
mtDNA: Mitochondrial DNA
PCR: Polymerase chain reaction
