# Supplementary material for: Coexistence of two sympatric cryptic bat species in French Guiana: insights from genetic, acoustic and ecological data
Source: BMC Evol Biol. 2018 Nov 20;18:175. doi: 10.1186/s12862-018-1289-8 (PMC6247516; doi:10.1186/s12862-018-1289-8)
Supplement: Supplementary file 1 — Table S1. Table. List of external COI sequences used in this study. The sequence in bold corresponds to type-locality of P. rubiginosus, caught in MatoGrosso, Brazil compared to P. sp4 in De Thoisy et al. (2014). Table S2. Characteristics of microsatellite markers. Figure S3. Graphical representation of assignation probabilities for Pteronotus A and Pteronotus B sampled. Each individual (x axis) is represented by a vertical bar divided in two parts according to its assignation probability (y axis) in each of the two clusters. Figure S4. Graphical representation of the difference between the Conservative and the Relaxed approach for the detection of hybrids. Table S5. Pairwise Fst values between caves for each species and both sexes. Table S6. Life cycle of Pteronotus A and B. PRE = Pregnant, LAC = Lactating females, PLAC = Post-lactating females, NS = No status (non pregnant, non-lactating, non post-lactating females, and non breeding males). n(A): number of Pteronotus A (number of adult females in parentheses); n(B): number of Pteronotus B (number of adult females in parentheses); n: total number of Pteronotus. (DOCX 319 kb) [file 12862_2018_1289_MOESM1_ESM.docx]

**Additional file 1**

**Additional file S1**

Table: List of external COI sequences used in this study. The sequence in bold corresponds to type-locality of *P. rubiginosus*, caught in MatoGrosso, Brazil compared to *P. sp4* in De Thoisy et al (2014).


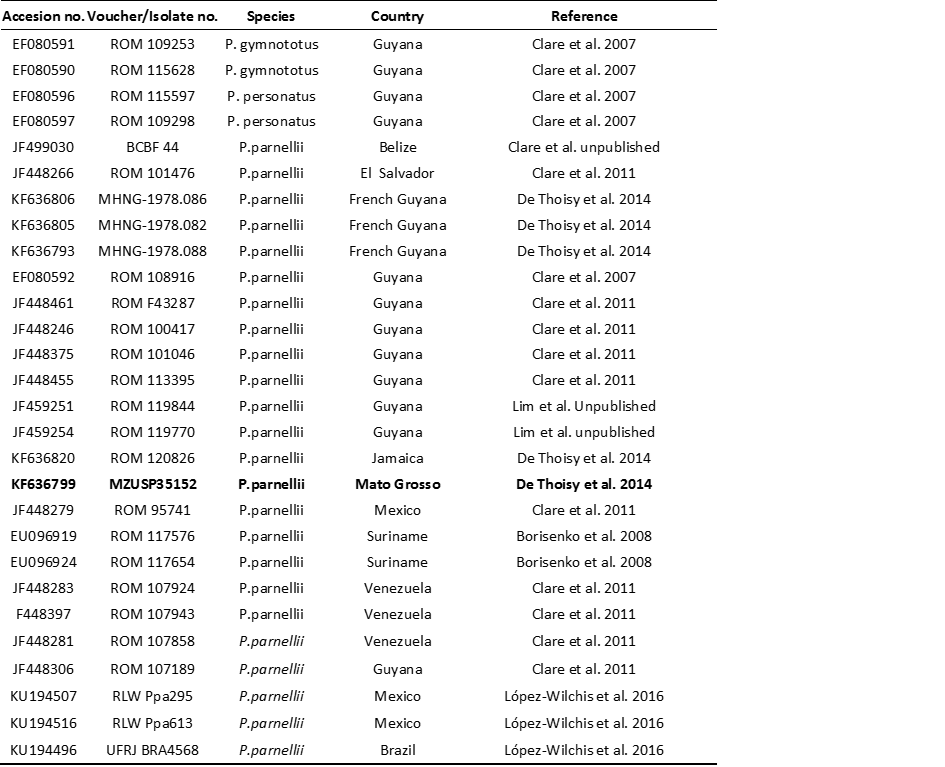


Additional file S2

Table: Characteristics of microsatellite markers.

**Additional file S3**

Graphical representation of assignation probabilities for *Pteronotus* A and *Pteronotus* B sampled. Each individual (x axis) is represented by a vertical bar divided in two parts according to its assignation probability (y axis) in each of the two clusters.


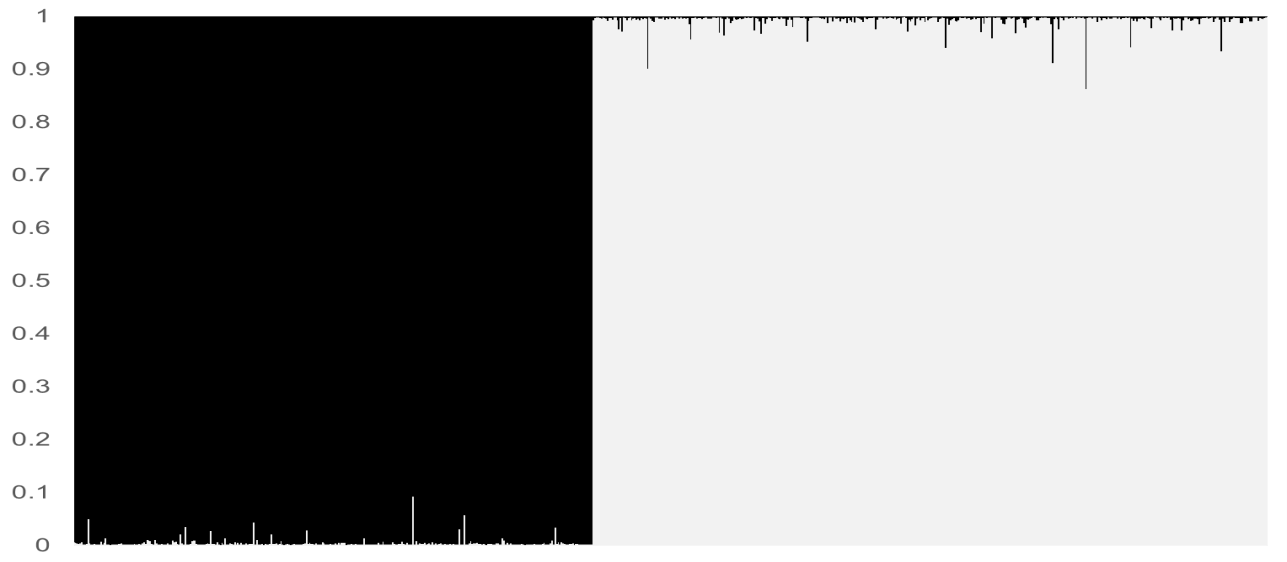


**Additional file S4**

Table: Pairwise *Fst* values between caves for each species and both sexes.

| **Females A** | | | |
| --- | --- | --- | --- |
|  | **PA** | **SC** | **MG** |
| **MA** | 0.095 | -0.018 | 0.009 |
| **PA** |  | 0.016 | 0.025 |
| **SC** |  |  | -0.004 |
| **Females B** | | | |
| **MA** | -0.001 | -0.003 | -0.002 |
| **PA** |  | -0.006 | -0.004 |
| **SC** |  |  | -0.010 |
| **Males A** | | | |
| **MA** | 0.006 | 0.000 | -0.008 |
| **PA** |  | **0.005*** | -0.011 |
| **SC** |  |  | -0.007 |
| **Males B** | | | |
| **MA** | 0.002 | 0.003 | -0.002 |
| **PA** |  | -0.001 | 0.000 |
| **SC** |  |  | 0.000 |

**Additional file S5**


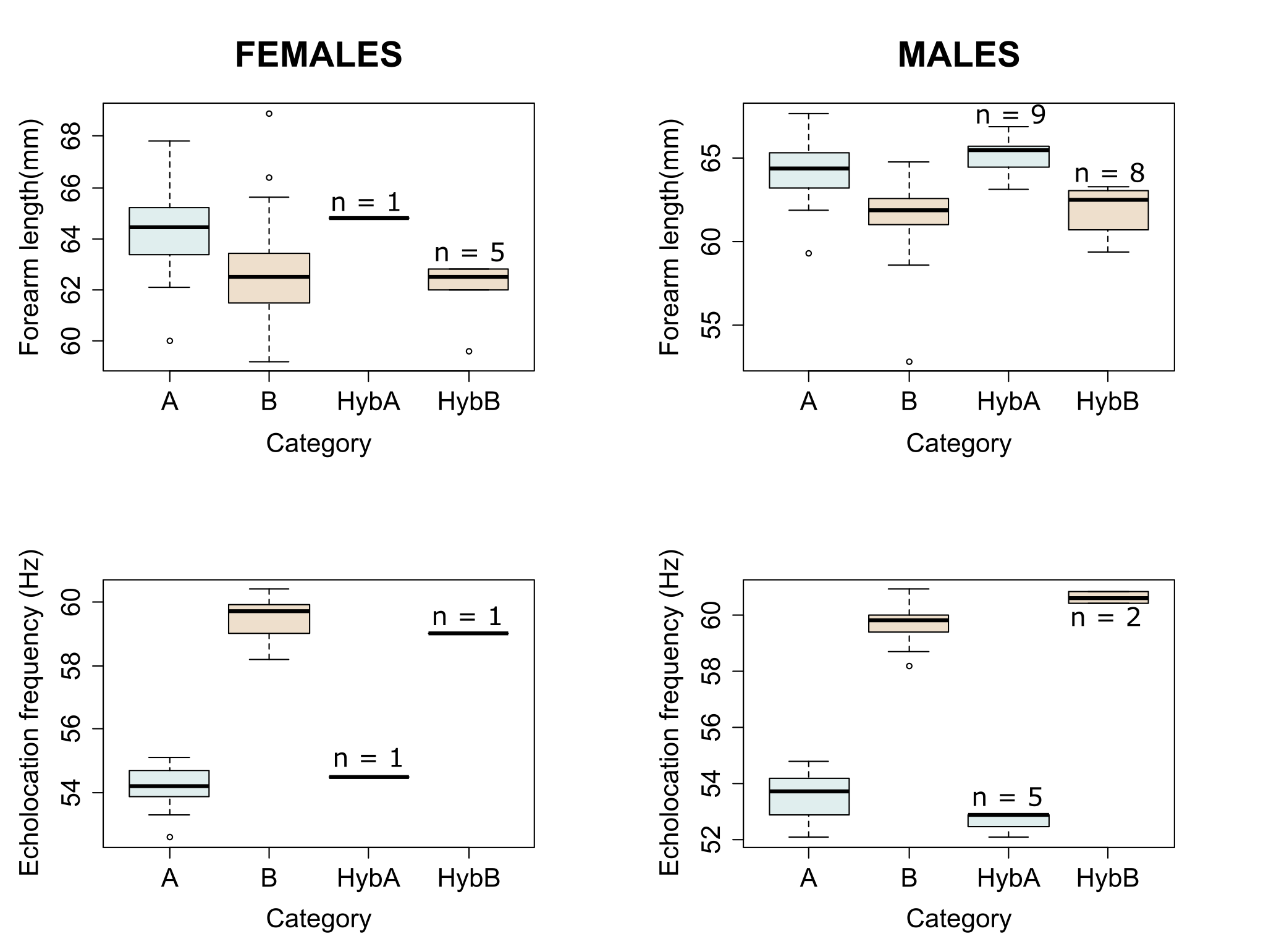


Figure: Forearm length (mm) and echolocation frequency (kHz) depending on the genetic status of individuals according to the relaxed approach (A = *Pteronotus* A, B = *Pteronotus* B, HybA = hybrids mostly assigned to cluster A by STRUCTURE, Hyb B = hybrids mostly assigned to cluster B by STRUCTURE). The number of individuals available for hybrid classes is indicated on the graph.

Globally, hybrids presented features similar to the the features observed in the cluster to which they had the higher assignment probability. The low number of individuals did not allow us to test reliably differences between pure individuals and hybrids.

**Additional file S6**

Table: Life cycle of *Pteronotus* A and B. PRE = Pregnant, LAC = Lactating females, PLAC = Post-lactating females, NS = No status (non pregnant, non-lactating, non post-lactating females, and non breeding males). n(A): number of *Pteronotus A* (number of adult females in parentheses); n(B): number of *Pteronotus B* (number of adult females in parentheses); n: total number of *Pteronotus*.

|  | ***Pteronotus A*** | | | | **n(A)** | ***Pteronotus B*** | | | | **n(B)** | **n** |
| --- | --- | --- | --- | --- | --- | --- | --- | --- | --- | --- | --- |
| **Status** | **PRE** | **LAC** | **PLA** | **NS** |  | **PRE** | **LAC** | **PLA** | **NS** |  |  |
| July | 2 | - | - | 117 | 119(24) | 27 | 15 | - | 95 | 137(74) | 256 |
| August | - | - | - | 1 | 1(0) | - | - | 2 | 5 | 7(3) | 8 |
| September | - | - | - | 121 | 121(24) | - | - | 12 | 89 | 101(29) | 222 |
| October | - | 1 | - | 83 | 84(24) | - | - | 19 | 159 | 178(71) | 262 |
| **Total** | **2** | **1** | **0** | **322** | **325(72)** | **27** | **15** | **33** | **348** | **423(177)** | **748** |
